# CCL5 Suppresses Klotho Expression *via* p-STAT3/DNA Methyltransferase1-Mediated Promoter Hypermethylation

**DOI:** 10.3389/fphys.2022.856088

**Published:** 2022-03-01

**Authors:** QiFeng Liu, ShaSha Li, LiXia Yu, XiaoYa Yin, Xi Liu, JianMing Ye, GuoYuan Lu

**Affiliations:** ^1^Department of Nephrology, The First Affiliated Hospital of Soochow University, Suzhou, China; ^2^Department of Nephrology, Affiliated Kunshan Hospital of Jiangsu University, Kunshan, China; ^3^Clinical Research & Lab Centre, Affiliated Kunshan Hospital of Jiangsu University, Kunshan, China

**Keywords:** C-C motif chemokine 5, Klotho, epigenetic, methyltransferase, STAT3

## Abstract

**Background:**

Enhanced inflammation and reduced Klotho are common features in chronic kidney disease (CKD). Inflammation induces DNA hypermethylation. This study assessed the performance of inflammatory marker C-C motif chemokine 5 (CCL5) in epigenetic regulation of Klotho expression.

**Methods:**

Fifty CKD patients and 25 matched controls were enrolled, and serum CCL5 level, sKlotho level, and DNA methylation were evaluated in these subjects. A renal interstitial fibrosis (RIF) model with CKD was induced in mice *via* unilateral ureteral obstruction (UUO) *in vivo* and human proximal tubular epithelial (HK-2) cells treated with CCL5 *in vitro*. 5-aza-2′-deoxycytidine (5-Aza), a DNA methyltransferase inhibitor was given to UUO mice. Hematoxylin and eosin (HE) and Masson trichrome staining were adopted to evaluate renal pathological changes. Methylation-specific PCR was performed to assess DNA methylation of Klotho promoter in the peripheral blood leucocytes (PBLs) from CKD patients and obstructive kidney from UUO mice. CCL5, Klotho, and DNA methyltransferases (DNMTs) were determined by ELISAs, immunofluorescence, or western blotting. HK-2 cells were exposed to CCL5 with or without 5-Aza and stattic, a p-signal transducer and activator of transcription 3 (STAT3) inhibitor, and expressions of p-STAT3, DNMT1, and Klotho were determined by western blotting.

**Results:**

CCL5 upregulation concomitant with Klotho downregulation in serum and global DNA methylation in PBLs were observed in CKD samples. UUO contributed to severe renal interstitial fibrosis and enhanced expressions of fibrotic markers. Moreover, UUO increased the CCL5 level, induced Klotho promoter methylation, suppressed Klotho level, activated p-STAT3 signaling, and upregulated DNMT1 level. A similar observation was made in HK-2 cells treated with CCL5. More importantly, 5-Aza inhibited UUO-induced Klotho hypermethylation, reversed Klotho, downregulated p-STAT3 expressions, and ameliorated RIF *in vivo*. The consistent findings *in vitro* were also obtained in HK-2 cells exposed to 5-Aza and stattic.

**Conclusion:**

The CCL5/p-STAT3/DNMT1 axis is implicated in epigenetic regulation of Klotho expression in CKD. This study provides novel therapeutic possibilities for reversal of Klotho suppression by CKD.

## Introduction

Klotho is a newly identified kidney-protective factor (Kuro-o et al., 1997) with various renoprotective actions that include inhibition of inflammation and renal interstitial fibrosis (RIF) (Hum et al., 2016; Maique et al., 2020). Because it is produced primarily by the kidney (Hu et al., 2016a), unsurprisingly, its expression is significantly reduced in chronic kidney disease (CKD; Shimamura et al., 2012; Wang et al., 2018). Klotho expression is downregulated in CKD, and therefore anti-fibrotic effects conferred by Klotho are remarkably abolished. Thus, Klotho deficiency is implicated in the initiation and development of RIF (Sugiura et al., 2012; Liu et al., 2017; Takenaka et al., 2017; Li et al., 2019a). It is reasonable that preventing Klotho decline will suppress RIF progression, which represents a potential strategy for CKD treatment (Neyra and Hu, 2017; Zou et al., 2018) as verified in our recent study (Li et al., 2018). Thus far, causes of Klotho loss remain under intense investigation. Emerging studies have shown epigenetic abnormalities in the Klotho promoter, such as DNA methylation, which dramatically contribute to Klotho deficiency (Azuma et al., 2012; Yin et al., 2017; Li et al., 2019b). Because the Klotho promoter contains rich CG sequences or CpG islands and is prone to hypermethylation, Klotho expression is consequently silenced at the transcript level (Azuma et al., 2012). Indeed, a growing number of preclinical or clinical studies have demonstrated the state of Klotho promoter methylation is closely linked to the extent of kidney Klotho loss in CKD (Sun et al., 2012; Chen et al., 2013). Therefore, DNA methylation in the Klotho promoter is proposed to be an important mechanism responsible for Klotho deficiency and a potential therapeutic target in CKD (Kale et al., 2021).

Enhanced inflammation is a characteristic of CKD patients and is involved in the development of CKD (Kooman et al., 2017; Black et al., 2019). Indeed, an increase of inflammatory markers, including c-reactive protein, tumor necrosis factor α, and interleukin-6, have been validated and predicted adverse kidney outcomes in CKD population (Pruijm et al., 2012; Amdur et al., 2016; Arnold et al., 2017). Furthermore, inflammation is inversely associated with the Klotho level (Oh et al., 2015; Ma et al., 2020b), which suggests a potential link between increased inflammation and Klotho deficiency. In fact, previous studies have demonstrated inflammation directly reduces Klotho expression by inhibiting Klotho gene transcription, although the mechanism behind this is not fully understood (Zhou et al., 2017; Sedighi et al., 2019). It has been reported that inflammation induces various epigenetic modifications in DNA and subsequently alters target gene expression (Stenvinkel et al., 2007). Whether inflammation regulates Klotho expression through DNA methylation remains to be investigated. C-C motif chemokine 5 (CCL5) is a potent proinflammatory factor that is remarkably upregulated in fibrotic kidneys (Zhang et al., 2019) and exerts a pathological role during the induction and progression of RIF (Zhang et al., 2012). CCL5 induces DNA methylation and influences the expression of target genes in tumors (Wang et al., 2017). It is unclear whether this mechanism acts in fibrotic kidney diseases.

Here, we postulated that CCL5 epigenetically mediated Klotho expression and the underlying mechanism was associated with CCL5-induced DNA methylation. Thus, we performed the current study to test or validate our hypothesis in CKD subjects, mice with unilateral ureter obstruction (UUO) induced-RIF, and CCL5-treated human proximal tubular epithelial (HK-2) cells. Our study will reveal a novel epigenetic regulation of inflammation-induced Klotho downregulation and a possible therapeutic target to treat RIF.

## Materials and Methods

### Clinical Study

This cross-sectional study was reviewed and approved by the ethics committee of Kunshan First People’s Hospital Affiliated to Jiangsu University. The study was conducted in accordance with the Declaration of Helsinki and informed written consent was provided by subjects. CKD was diagnosed by the criteria of KDIGO and was classified in accordance with the estimated glomerular filtration rate (eGFR; Stevens et al., 2013). eGFR was calculated by the CKD—Epidemiology Collaboration equation (Stevens et al., 2011). Twenty-five healthy volunteers matched with age and sex were recruited as controls based on the exclusion criteria. The study was performed from September 2019 to February 2020. The exclusion criteria were as follows: acute kidney disease (AKI) or AKI on CKD, infectious diseases, which included respiratory, digestive, and urinary tract infections, tumor or cancer, use of immunosuppressants, aged <18 years, and a history of renal replacement therapy or organ transplantation. Baseline demographic and clinical data, such as age, gender, medicines, and history of CKD, were recorded at study enrollment. Laboratory parameters, which included hemoglobin (HGB), calcium, inorganic phosphate, blood urea nitrogen (Bun), and serum creatinine (Scr) were measured. The serum levels of CCL5, a marker of inflammation, and sKlotho were detected by ELISAs (E-EL-H6006 & E-EL-H5451c, Elabscience) in accordance with the manufacturer’s instructions.

Peripheral whole blood from CKD patients and healthy controls was obtained and genomic DNA was extracted using a Quick-DNA™ Miniprep Plus Kit (D4068, ZYMO Research). DNA and serum were immediately stored at −80°C for further analyses.

### *In vivo* Experiments

#### Induction of RIF

Twenty-four C57BL/6 mice (8 weeks old, weighing 20–25 g) were purchased from Joinn Laboratories Co., Ltd. (Suzhou, China). They were housed in a temperature- and light-controlled room and given freely standard diet and water. The mice were divided into: sham, UUO, 5-aza-2′-deoxycytidine (5-Aza) treatment, and UUO plus 5-Aza treatment group (*n* = 6, each). RIF was established *via* UUO as described previously (Li et al., 2019a). Demethylating agent 5-Aza (0.35 mg/kg, ST1047, Beyotime, China) was injected intraperitoneally every alternate day. Mice in the sham group received the same procedure, but without ureter obstruction. After 10 days, mouse kidneys from UUO side were removed for subsequent analyses. All procedures were approved by the Animal Care and Use Committee of Affiliated Kunshan Hospital of Jiangsu University and in agreement with ARRIVE guidelines (Kilkenny et al., 2010).

### Renal Histopathological Analysis

All mice were sacrificed at 10 days under general anesthesia. Kidneys were removed, immediately fixed in 4% paraformaldehyde, and then embedded in paraffin for pathological examination. The prepared sections were stained with hematoxylin and eosin (HE) and Masson trichrome. The degree of RIF was assessed by Masson trichrome, and semi-quantitatively measured based on the ratio of collagen deposition under light microscope using Image-Pro Plus 6.0 software.

### Immunofluorescence

Dewaxed kidney tissue sections were rehydrated, heated for 20 min in a microwave for antigen retrieval, and then incubated with immunohistochemical serum blocking agent (GEPbio, 317615) for 30 min at 37°C to block non-specific staining. Subsequently, the sections were incubated with a rabbit anti-Klotho antibody (ab181373, Abcam) and rat anti-CCL5 antibody (NB120-10394, NOVUS) overnight at 4°C. The next day, the sections were washed with PBS for three times and then incubated with an Alexa Fluor 555-labeled anti-rabbit second antibody (ab150078, Abcam) and Alexa Fluor 488-labeled anti-rat second antibody (ab150157, Abcam) at 37°C for 30 min. Then, cell nuclei were counterstained with 4′,6-diamidino-2-phenylindole (DAPI, GEPbio, 721621) for 3 min. Finally, the sections were washed with PBS again and mounted with anti-fade medium (GEPbio, 717615). Stained sections were examined and imaged under a fluorescence microscope. Mean fluorescence intensity (MFI) of each section was analyzed by Image J software.

### Western Blotting Analysis

Total proteins of frozen kidney tissues and harvested cells were extracted with radioimmunoprecipitation buffer (R0020, Solarbio) with a protease inhibitor cocktail (P6730, Solarbio). After quantification with a BCA protein assay kit (No. 23227, Thermo Fisher), protein (20 μg) was separated by 8% or 10% SDS-PAGE and then transferred to a PVDF membrane. The membranes were blocked in 5% non-fat milk at room temperature (RT) for 1 h and then incubated with primary antibodies overnight at 4°C. Primary antibodies used were shown below: anti-Klotho (ab181373, Abcam; sc-515942, Santa Cruz), anti-DNA methyltransferase (DNMT) 1 (#5032, CST), anti-DNMT3a (#3598, CST), anti-DNMT3b (#67259, CST), anti-p-STAT3 (ab76315, Abcam), anti-α-smooth muscle actin (α-SMA; #14968, CST), anti-E-cadherin (E-cad; #14472, CST), and anti-GAPDH (ab181603, Abcam). The next day, membranes were incubated with HRP-labeled secondary antibodies (sc-2357 & sc-2005, Santa Cruz) for 1 h at RT. Immunoblot signals were detected and then analyzed by Image J software with normalization to GAPDH.

### Methylation-Specific PCR

Genomic DNA from human leukocytes or renal mouse tissue was extracted using a Quick-DNA™ Miniprep Plus Kit (D4068, ZYMO RESEARCH Corp.). An EZ DNA Methylation-Gold™ Kit (D5006, ZYMO RESEARCH Corp.) was used to convert unmethylated cytosines to uracil, so that the DNA could be analyzed by PCR amplification. Methylated Klotho, Unmethylated Klotho, and Input were detected using ZymoTaq™ PreMix (E2004, ZYMO RESEARCH Corp.; Yin et al., 2017). Human Klotho promoter (−315/−99) and Mouse Klotho promoter (+497/+685) were analyzed by methylation-specific PCR (MSP), because these positions were rich in CpG islands near transcription starting site (Sun et al., 2012; Yin et al., 2017). Primers are listed in Supplementary Table 1 in accordance with the literature (Sun et al., 2012; Yin et al., 2017). PCR products were analyzed by 1.2% (*w*/*v*) agarose gel electrophoresis and densitometry was conducted using a ChemStudio system (Analytik Jena AG, Germany). The quantification is presented as the ratio of methylated or unmethylated PCR products to the total PCR products.

### *In vitro* Experiments

#### Cell Culture

HK-2 cells were purchased from the National Collection of Authenticated Cell Cultures (Shanghai, China) and cultured in DMEM-F12 supplemented with 10% fetal bovine serum at 37°C with 5% CO_2_. To investigate the effect of CCL5, HK-2 cells were exposed to 0.5 or 1 μg/ml CCL5 (sc-4,637, Santa Cruz) for 24 h. To verify the effect of STAT3, STAT3 inhibitor stattic (10 μM, ab120952, Abcam) was applied. To verify the effect of DNMT1, DNMT1 inhibitor 5-Aza (10 μM) was applied. Cells were harvested for western blotting analysis.

### Statistical Analysis

Results are presented as the mean ± SD or median with 25th–75th percentile of independent experiments. SPSS software 23.0 and GraphPad 9.0.3 were used for data analysis. ANOVA or non-parametric test was adopted for data analysis among groups. *p* < 0.05 indicated significant difference and ns indicated as no significant difference.

## Results

### Clinical Samples

Fifty CKD patients at stages 1–5 and 25 matched controls were enrolled. The clinical characteristics are listed in Supplementary Table 2. The serum Klotho level was decreased, while CCL5 level was increased in CKD patients compared with controls (Supplementary Table 2, Supplementary Figures 1a,b). Furthermore, similar changes in Klotho and CCL5 levels persisted during progression of kidney dysfunction (Supplementary Figures 1a,b). A negative correlation between Klotho and CCL5 levels was also observed (Supplementary Figure 1c). Previous reports have shown that peripheral blood leucocytes (PBLs) in CKD patients exhibit the characteristics of global DNA hypermethylation (Stenvinkel et al., 2007; Chen et al., 2019a). A recent study showed that DNA methylation in the Klotho promoter of PBLs is strongly associated with DNA methylation in the renal Klotho promoter, which predicts renal Klotho promoter methylation and subsequently affects Klotho expression (Chen et al., 2013). Thus, the degree of DNA methylation was also determined in the Klotho promoter of PBLs. Notably, the Klotho promoter in PBLs was heavily hypermethylated in CKD compared with that in PBLs in controls (Supplementary Figure 1d). This observation indicated that abnormal epigenetics were involved in Klotho suppression.

### *In vivo* Experiments

#### Decreased Renal Klotho and Increased CCL5 Expression in UUO Mice

After UUO surgery, renal histology results obtained by hematoxylin and eosin staining showed increases in cell infiltration, degeneration and necrosis of epithelial cells, interstitial edema, and renal tubular dilation (Supplementary Figure 2a). Masson staining also showed that the degree of RIF was enhanced dramatically (Supplementary Figures 2a,b). Accordingly, a fibrotic marker, α-SMA was upregulated accompanied by the downregulation of the epithelial marker, E-cad (Supplementary Figures 3b,c). Furthermore, renal Klotho expression was strongly downregulated in both immunofluorescence and western blotting analyses (Supplementary Figures 3a,b,e) and the CCL5 level was upregulated significantly as shown by immunofluorescence (Supplementary Figure 3a). These data suggested CCL5 was negatively associated with Klotho, in accordance with the findings from CKD subjects.

#### Decreased Renal Klotho Expression Is Associated With DNA Methyltransferase 1-Induced Hypermethylation of Its Promoter in UUO Mice

Because Klotho is modulated epigenetically, we next analyzed the methylation status of the Klotho promoter. UUO resulted in renal Klotho deficiency showed by immunofluorescence and western blotting analyses (Supplementary Figures 3a,b,e) and severe methylation of the Klotho promoter showed by MSP assays (Supplementary Figures 3g,h). To clarify the relationship between Klotho expression and promoter methylation, DNMTs, which included DNMT1, DNMT3a, and DNMT3b, were analyzed. UUO induced dramatic DNMT1 upregulation, slight changes in DNMT3a and DNMT3b levels (Supplementary Figures 3b,f ). Meanwhile, DNMT1 elevation was concomitant with p-STAT3 activation and increased DNA methylation (Supplementary Figures 3b,d,f,g,h). Intriguingly, a specific inhibitor of DNMT1, 5-Aza, decreased p-STAT3 expression, attenuated DNA methylation and subsequently reversed the effect on Klotho expression (Supplementary Figures 3b,d–h). Furthermore, 5-Aza treatment ameliorated renal tubular and interstitial damage (Supplementary Figures 2a,b, 3b,c). Taken together, these data indicated that UUO increased CCL5 level, induced epigenetic DNA methylation, and followed by suppression of Klotho expression and induction of RIF.

### *In vitro* Experiments

#### Klotho Expression Is Downregulated in CCL5-Treated HK-2 Cells

To examine whether Klotho expression was downregulated by CCL5 in HK-2 cells, we detected Klotho expression by western blotting. HK-2 cells were incubated with CCL5 (0.5 μg/ml or 1.0 μg/ml) and then Klotho expression was evaluated. As shown in Supplementary Figure 4a, after CCL5 treatment, expression of Klotho protein in HK-2 cells was not decreased significantly at the dosage of 0.5 μg/ml; however, its expression was dramatically decreased at the dosage of 1.0 μg/ml. The similar findings were also shown in Supplementary Figures 4b,c. Together, our results revealed that CCL5 was capable of repressing Klotho expression in HK-2 cells.

#### Increased DNMT1 Is Associated With Repressed Klotho Expression in CCL5-Treated HK-2 Cells

Since Klotho is regulated epigenetically *in vivo* study (Supplementary Figures 3g,h), we further investigate the potential mechanism *in vitro*. Based on the observations from *in vivo* study, DNMT1 was an important player in Klotho methylation, thus we analyzed the changes of DNMT1 in HK-2 cells. CCL5 treatment induced dramatic upregulation of DNMT1 at the dosage of 1.0 μg/ml, accompanied by Klotho repression (Supplementary Figure 4a), suggesting that DNMT1 correlated inversely Klotho. Interestingly, a specific inhibitor of DNMT1—5-Aza suppressed DNMT1 expression and subsequently reversed CCL5-triggered Klotho downregulation (Supplementary Figure 4b). This indicated that DNMT1 played an important role in inducing Klotho suppression, similar to the observation from *in vivo* study, and the underlying mechanism possibly was associated with DNMT1 induced DNA methylation of Klotho promoter.

#### DNMT1 Expression Is Regulated by the pSTAT3 Signaling Pathway in CCL5-Treated HK-2 Cells

DNMT1 was a major player in inducing methylation of Klotho promoter *in vivo* study and its expression was increased significantly in HK-2 cells by CCL5 treatment. To explore the regulatory mechanism of CCL5-DNMT1 interplay, we examined p-STAT3 signaling pathways because pSTAT3 signaling is both downstream of CCL5 and upstream of DNMT1. Accordingly, we firstly found that CCL5 remarkably elevated p-STAT3 and DNMT1 expression, indicating that CCL5 activated p-STAT3 signaling pathway (Supplementary Figure 4a). Intriguingly, an inhibitor of p-STAT3, stattic, largely decreased p-STAT3 and DNMT1 expression and reversed Klotho expression (Supplementary Figure 4c). This suggested that CCL5 triggered p-STAT3 signaling and mediated DNMT1 upregulation in HK-2 cells, demonstrating the critical role of p-STAT3 or DNMT1 in Klotho regulation in HK-2 cells. Notably, stattic did not upregulated significantly Klotho expression in HK-2 cells in the presence of CCL5 treatment (Supplementary Figure 4c).

## Discussion

In this study, we found that Klotho downregulation in CKD patients and UUO mice was associated with DNA methylation. CCL5 and its downstream STAT3 signaling was responsible for Klotho epigenetic alterations, which suggested that CCL5 regulated Klotho expression *via* a STAT3-DNMT1-dependent mechanism.

Many studies have documented that kidney injury is aggravated in the absence of Klotho, while the injury is ameliorated in the presence of Klotho, which corroborates that Klotho is a novel kidney-protective protein (Jin et al., 2016; Li et al., 2018; Takenaka et al., 2020; Yuan et al., 2022). Unsurprisingly, if Klotho expression is decreased or suppressed, its kidney-beneficial effects are abrogated. As a result, Klotho deficiency makes the kidney vulnerable to various insults, thus contributes to the progression of kidney diseases (Sugiura et al., 2012; Jin et al., 2016; Li et al., 2018). Therefore, retarding the decline in Klotho expression or upregulation of endogenous Klotho are plausible strategies to eliminate or suppress kidney disorders (Neyra et al., 2020a). In agreement with this, an increased Klotho level has been associated with less adverse kidney outcomes in human studies (Drew et al., 2017; Liu et al., 2018; Yang et al., 2020). However, the regulatory mechanism of Klotho deficiency in CKD remains unclear and warrants intensive investigation (Kale et al., 2021). Only if regulatory mechanisms are better understood, potential strategies can be developed to increase the Klotho level for the treatment of kidney diseases with Klotho deficiency. Declined kidney functions, inflammation, reactive oxygen species, uremic toxins, endoplasmic reticulum stress (ERS) and the renin–angiotensin system have been reported to act as negative regulators of Klotho expression, which contribute to the suppression of Klotho expression *via* different mechanisms (Moreno et al., 2011; Hu et al., 2016a; Raeisi et al., 2016; Ma et al., 2020a). However, there are increasing evidences suggesting that epigenetic modifications of the Klotho gene, such as promoter hypermethylation, play a critical role during this pathological process, especially in early or intermediate CKD stages (Azuma et al., 2012; Chen et al., 2013). Because of the potential high risk of renal biopsy in advanced CKD patients, the level of Klotho promoter hypermethylation in PBLs was measured in this study instead of performing a renal biopsy, considering that it predicts renal Klotho promoter methylation with high sensitivity and specificity (Chen et al., 2013). In agreement with this, we observed a close correlation between methylation in the Klotho promoter of PBLs and Klotho protein. Promoter methylation of Klotho blocked Klotho expression and contributed to systematic suppression of Klotho, while suppression of DNA methylation within the Klotho promoter significantly restored Klotho expression, which suggests that an abnormality in DNA methylation represents a novel therapeutic target for a disease state with Klotho deficiency (Chen et al., 2016; Hu et al., 2016b; Li et al., 2019b).

Inflammation has a critical and decisive role in the initiation and development of kidney diseases. The majority of kidney disorders feature enhanced inflammation concomitant with decreased Klotho expression. CCL5 is a major mediator and biomarker of inflammation and it recruits monocytes, macrophages, T cells, and eosinophils to the sites of injured kidneys. Indeed, a recent study showed that the CCL5 level was significantly associated with the intensity of RIF and CCL5 was identified as a potential intervention target for RIF (Zhang et al., 2019). Our data from human CKD showed that the CCL5 level was elevated significantly, while the Klotho level was significantly decreased as eGFR declined. CCL5 was inversely associated with Klotho. This phenomenon was further demonstrated in our CKD model, which indicates that CCL5 as a major regulator of inflammation may downregulate Klotho expression.

Inflammation induces epigenetic modifications, primarily DNA methylation, and contributes to changes in target protein expression. Similarly, CCL5 was also reported to trigger DNA methylation and block target protein expression in several tumors. Therefore, we postulated that CCL5 induced Klotho promoter methylation and subsequently downregulated Klotho expression. DNA methylation is driven by several DNMTs that include DNMT1, DNMT3a, and DNMT3b. Previous study reported that DNMT1 was notably upregulated in fibrotic kidneys compared with other DNMTs, and DNMT1 inhibition effectively mitigated structural and functional damage in kidney from several CKD models (Sun et al., 2012; Yin et al., 2017; Gao et al., 2022), supporting DNMT1 is emerging as a player and a therapy target in CKD (Zhang et al., 2017). Moreover, specific inhibition of DNMT1 suppressed DNA methylation-induced Klotho loss, which indicated the critical role of DNMT1 in this process of DNA methylation (Chen et al., 2016; Hu et al., 2016b; Li et al., 2019b). That is to say, DNMT1 is potential link of DNA methylation with Klotho epigenetic loss. In accordance with this, we observed that the CKD model in mice displayed increased methylation of the Klotho promoter and Klotho loss, followed by remarkable elevation of the DNMT1 level. Interestingly, as a specific inhibitor of DNMT1, 5Aza also reversed suppression of Klotho expression in HK-2 cells treated with CCL5. These results support the crucial role of DNMT1 in CCL5-induced Klotho promoter methylation.

STAT3 is a STAT family member that has essential roles in embryonic development, cell survival, inflammation, fibrosis, and cancer metastasis (Takeda et al., 1997; Yu et al., 2009; Hillmer et al., 2016). STAT3 is located in the cytoplasm as an inactive form, but is stimulated by diverse upstream activators (Chen et al., 2019b). After activation, STAT3 is phosphorylated at tyrosine 705 and then p-STAT3 translocates from the cytoplasm to the nucleus and binds to its specific sequence in DNA to modulate target gene transcription (Chen et al., 2019b). E-Cad, a-SMA, and other kidney fibrotic molecules were also affected by p-STAT3 signaling *in vivo* and *in vitro* studies (Pang et al., 2010; Zheng et al., 2019), and p-STAT3 pathway plays a crucial role in the progression of various fibrotic kidney disease (Pace et al., 2019; Zheng et al., 2020). CCL5 is as an upstream regulator of STAT3 signaling and it interacts with C-C chemokine receptor type 5 to trigger its downstream STAT3 signaling (Zhou et al., 2016; Xiang et al., 2019). DNMT1 is a downstream target gene of STAT3 signaling in tumors. Thus, activation of STAT3 signaling upregulates DNMT1 expression and leads to DNA methylation (Zhang et al., 2006; Wang et al., 2017). Previous studies have demonstrated upregulation of CCL5 and STAT3 expression in kidney fibrosis, and their levels are positively associated with the degree of RIF (Bienaime et al., 2016; Pace et al., 2019; Zhang et al., 2019). Thus, CCL5/STAT3 signaling is involved in renal fibrotic diseases and is proposed to be a feasible therapeutic target for RIF. However, whether CCL5/STAT3 signaling affected Klotho promoter methylation in RIF remained unclear. Therefore, we examined changes in signal molecules of the CCL5/STAT3/DNMT1 pathway. We found that CCL5 treatment upregulated both p-STAT3 and DNMT1 levels, but p-STAT3 inhibition—stattic largely abolished upregulation of DNMT1 expression. Intriguingly, CCL5/STAT3/DNMT1 blockade restored Klotho expression *in vivo* study, which suggests that such signaling is possibly implicated in the modulation of Klotho expression. Collectively, our results revealed a novel epigenetic mechanism of Klotho deficiency and therapeutic target to rescue Klotho expression in RIF.

In fact, many preclinical studies have elaborated restoration of epigenetic abnormality of Klotho expression provided new therapeutic performance for diverse etiologies of kidney diseases including CKD (Hu et al., 2016b; Li et al., 2019b; Xia and Cao, 2021; Kale et al., 2022). This indicated that epigenetic regulation of Klotho possibly represents potential directions and contributes to novel therapeutic development for CKD (Yin et al., 2017; Li et al., 2019b; Kale et al., 2022). Consequently, well-designed clinical studies with DNA demethylation agents are needed to test or validate the results. Yet, it must be kept in mind that epigenetic modification may be disease-specific, and more attention should be paid to the safety and effectiveness of these agents before clinical translation.

### Limitations

There are a number of limitations to be addressed in this study. Firstly, there is no established ELISA assay for circulating Klotho measurement until now (Neyra et al., 2020b). There are apparent variations among several available assays, and these fluctuations inevitably affect the interpretation of our results (Neyra et al., 2020b). Secondly, it remains unclear that the kidney-protective effects of Klotho are mediated by membranous Klotho or systemic Klotho. We postulated the reno-protection of Klotho possibly might be mediated by systemic Klotho, because previous studies reported that exogenous Klotho protein therapy inhibited RIF in Klotho deficiency condition (Takenaka et al., 2017; Li et al., 2019a). Thirdly, stattic combination with CCL5 failed to furtherly elevate Klotho expression compared with stattic or CCL5 treatment *in vitro* study. Perhaps STAT3 signaling is not the only way to act in DNMT1-induced Klotho methylation. Because Klotho expression was also controlled by other epigenetic mechanisms, such as histone acetylation (HDAC), transcription factors (TFs), and non-coding RNA (Kale et al., 2021; Xia and Cao, 2021). A recent study reported that renal HDAC3 expression was preferentially increased in UUO mice and selective inhibition of HDAC3 derepressed Klotho expression and alleviated RIF (Chen et al., 2021). Previous study demonstrated that there were several binding sites for specific protein 1 (Sp1) within Klotho promoter (Azuma et al., 2012), and Sp1 overexpression or knockdown enhanced Klotho or suppressed Klotho expression induced by TGF-β1 in HK-2 cells, indicating that Sp1 regulated epigenetically Klotho expression (Li et al., 2020). In addition, other TFs, such as TGFβ1, nuclear factor kB (NF-kB), and activating transcription factor 3 (ATF3), are also involved in Klotho transcription inhibition (Moreno et al., 2011; Yin et al., 2017; Delitsikou et al., 2020). Therefore, the impacts of these regulators on Klotho expression after CCL5 treatment should not be excluded entirely and their specific roles should be examined and clarified in future study. This interesting phenomenon unravels the more complicated regulatory network of Klotho expression than expected. Finally, we only provided association and implication regarding the interaction of inflammation, epigenetics, and Klotho in CKD. More studies are still acquired to test or validate the findings by using small interfering RNA or specific antibody of CCL5 due to a lack of direct evidence regarding the relationship of CCL5 and Klotho in this study and there was still a long way before clinical translation in future.

In conclusion, we demonstrated that CCL5 mediates Klotho deficiency epigenetically, which involves activation of STAT3/DNMT1 signaling, and targeting the CCL5/STAT3/DNMT1 axis may be a potential therapeutic strategy to recover Klotho under Klotho-deficient states.

## Data Availability Statement

The original contributions presented in the study are included in the article/**Supplementary Material**, further inquiries can be directed to the corresponding authors.

## Ethics Statement

The studies involving human participants were reviewed and approved by the ethics committee of Kunshan First People’s Hospital Affiliated to Jiangsu University. The patients/participants provided their written informed consent to participate in this study. The animal study was reviewed and approved by the ethics committee of Kunshan First People’s Hospital Affiliated to Jiangsu University. Written informed consent was obtained from the individual(s) for the publication of any potentially identifiable images or data included in this article.

## Author Contributions

QL: conceptualization, methodology, investigation, data analysis, and writing. SL: visualization, software, data analysis, and writing. LY: investigation and data curation. XY: resources, methodology, and validation. XL: data curation and validation. JY: visualization, design, review, and editing. GL: conceptualization, design, supervision, review, and editing. All authors contributed to the article and approved the submitted version.

## Funding

This study was supported by the Social Development Foundation of Kunshan (KS1933) and Scientific Research Project—Jiangsu Commission of Health (Z2020004).

## Conflict of Interest

The authors declare that the research was conducted in the absence of any commercial or financial relationships that could be construed as a potential conflict of interest.

## Publisher’s Note

All claims expressed in this article are solely those of the authors and do not necessarily represent those of their affiliated organizations, or those of the publisher, the editors and the reviewers. Any product that may be evaluated in this article, or claim that may be made by its manufacturer, is not guaranteed or endorsed by the publisher.
