# Supplementary material for: CCL5 Suppresses Klotho Expression via p-STAT3/DNA Methyltransferase1-Mediated Promoter Hypermethylation
Source: Front Physiol. 2022 Mar 1;13:856088. doi: 10.3389/fphys.2022.856088 (PMC8922032; doi:10.3389/fphys.2022.856088)
Supplement: Supplementary file 1 [file Data_Sheet_1.docx]

**Table 1 MSP primers**

| Primer name | Sequences |
| --- | --- |
| hKL/M-F | AAAGAGAATGAATTTGAGC GTTTAC |
| hKLM-R | ACTCCGCTAACAATAATTACCTACG |
| hKL/unM-F | AAGAGAAT GAATTTGAGTGTTTATGA |
| hKL/unM-R | TCCACTAACAATAATTACCTACAAA |
| hInp-F | CCAACTCCAAATCCCCTCTCTAT |
| hInp-R | TGATTAATTTAGATTGGGTTTAGAGAA GGA |
| mKL/M-F | GGTATCGCGGGTATTTTTAATC |
| mKLM-R | CGACATAAT CCC TAAAATAATCGAC |
| mKL/unM-F | TTAATGGTATTGTGGGTATTTTTAATTG |
| mKL/unM-R | CAACATAATCCC TAAAATAATCAAC |
| mInp-F | TAGTTTTAGGAAGGTAAAGGGAGTG |
| mInp-R | AAATCCCAAAAAAAACACAACAAA |

**Table 2 Comparison of** **clinical data with CKD and controls**

| Variables | CKD (n = 50) | Controls (n = 25) | *P value* |
| --- | --- | --- | --- |
| Ages | 53.6±15.8 | 46.9±12.2 | 0.066 |
| Male (%) | 29(58.0) | 15(60.0) | 0.868 |
| Scr（μmol/L） | 328.3±252.8 | 83.2±16.0 | < 0.001 |
| BUN（mmol/L） | 15.0±9.1 | 5.8±1.5 | < 0.001 |
| HGB（g/L） | 109.6±25.8 | 143.8±12.4 | < 0.001 |
| CCL5 (pg/mL) | 59.7±10.3 | 44.4±7.3 | < 0.001 |
| sKlotho (ng/mL) | 0.51(0.41,0.60) | 0.86(0.69,1.10) | < 0.001 |

Abbreviations: Scr, serum creatinine; BUN, blood urea nitrogen; HGB, hemoglobin; Data are presented as mean ± SD for normally distributed variables, otherwise median with 25^th^ -75th percentile.
