# Supplementary material for: CCL5 Suppresses Klotho Expression via p-STAT3/DNA Methyltransferase1-Mediated Promoter Hypermethylation
Source: Front Physiol. 2022 Mar 1;13:856088. doi: 10.3389/fphys.2022.856088 (PMC8922032; doi:10.3389/fphys.2022.856088)
Supplement: Supplementary file 2 [file Data_Sheet_2.docx]

**Figure 1 CKD patients displays sKlotho suppression, enhanced CCL5 level in serum and Klotho hypermethylation.** (a) Serum sKlotho levels in CKD patients and healthy controls detected by ELISA; (b) Serum CCL5 level in CKD patients and healthy controls detected by ELISA; (c) Correlation analysis of sKlotho and CCL5; (d) Representative MSP of Klotho promoter methylation from CKD patients and healthy controls (Four randomly selected samples were shown). Data are presented as the mean ± SD or median with 25th -75th percentile (n = 6). * p<0.05, ** denotes p<0.01.

**Figure 2** **5-Aza alleviates renal fibrosis induced by UUO in mice.** (a) Representative micrographs of HE and Masson trichrome (scale bar, 100 μm). (b) Semiquantitative data of fibrosis area. Data are presented as the mean ± SD (n = 6). ** p<0.01.

**Figure 3 5-Aza attenuated UUO-triggered renal fibrosis and DNMT1-mediated Klotho hypermethylation in mice**. (a) Representative immunofluorescence images showing Klotho and CCL5 expression from Sham, UUO, 5-Aza and UUO plus 5-Aza mice. Klotho expression: red labelling; CCL5 expression: green labelling; nuclei: blue labelling. Scale bars, 100 μm. MIF: Mean fluorescence intensity. (b) Representative images of α-SMA, E-cad, Klotho, p-STAT3, DNMT1, DNMT3a and DNMT3b were determined by western blotting. Fold expression of (c) α-SMA (c), E-cad(c), Klotho (e), p-STAT3(d), DNMT1 (f), DNMT3a (f) and DNMT3b (f). (g) Representative MSP of Klotho promoter methylation from Sham, UUO, 5-Aza and UUO plus 5-Aza mouse kidneys. (Two randomly selected samples were shown). Methylated (M), Unmethylated (UM) and Input control PCR products were analyzed by agarose gel electrophoreses. (h) Quantification of MSP products in Fig. 3g from all experimental mice. The results were presented as the percentile changes of M/UM over total PCR products. Data are presented as means ± SD. * p<0.05, ** denotes p<0.01; ns, no significance.

**Figure 4 CCL5 activated DNMT1-p-STAT3 and inhibited Klotho expression, but 5-Aza and stattic reversed CCL5-triggered Klotho downregulation in vitro.** (a) HK-2 cells were treated with 0.5 or 1 μg/mL CCL5 for 24 h. Expression of DNMT1, p-STAT3 and Klotho was examined by western blotting, and their relative expression levels. . (b) HK-2 cells were treated with 1 μg/mL CCL5 with or without 5-Aza (10 μM) for 24 h. Expression of DNMT1, p-STAT3 and Klotho was examined by western blotting, and their relative expression levels. (c) HK-2 cells were treated with 1 μg/mL CCL5 with or without stattic (10 μM) for 24 h. Expression of DNMT1, p-STAT3 and Klotho was examined by western blotting, and their relative expression levels.. Data are presented as the mean ± SD (n = 4). * p<0.05, ** p<0.01; ns, no significance.
