# Supplementary figures and images for: CCL5 Suppresses Klotho Expression via p-STAT3/DNA Methyltransferase1-Mediated Promoter Hypermethylation
Source: Front Physiol. 2022 Mar 1;13:856088. doi: 10.3389/fphys.2022.856088 (PMC8922032; doi:10.3389/fphys.2022.856088)

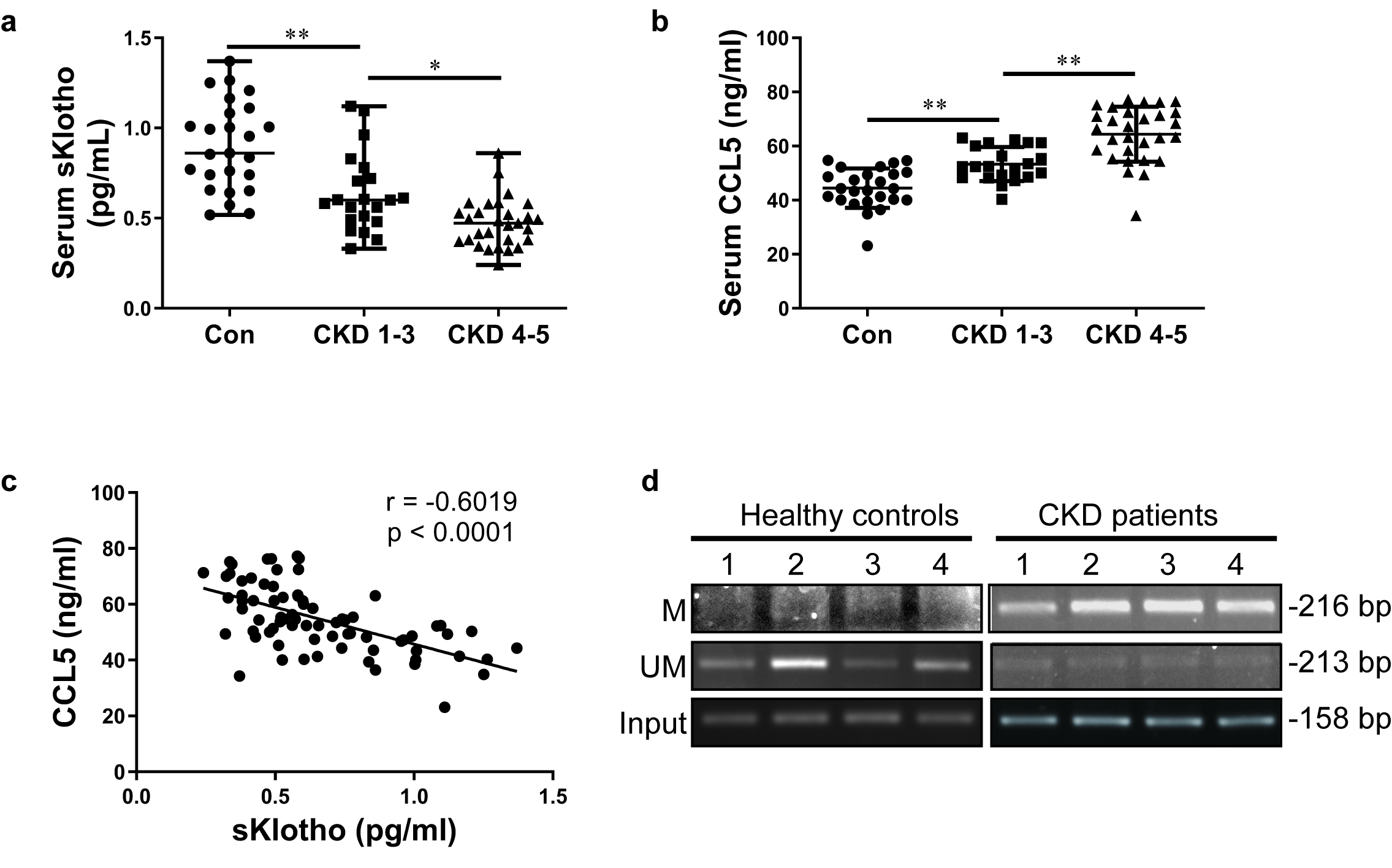

Supplement: Supplementary file 3 [file Image_1.tif]

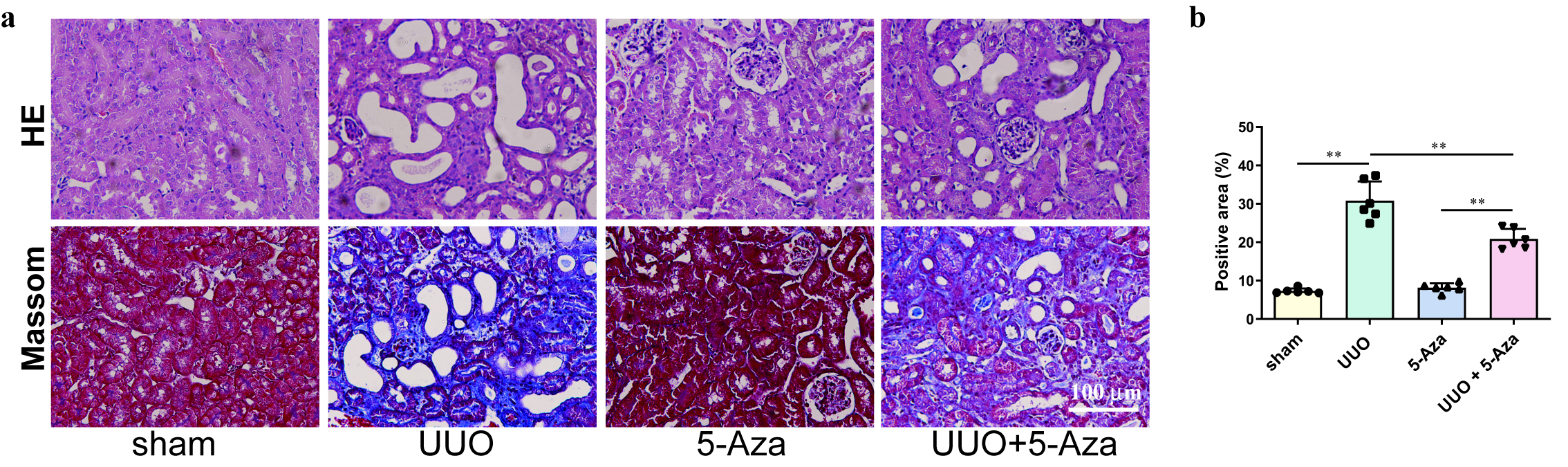

Supplement: Supplementary file 4 [file Image_2.tif]

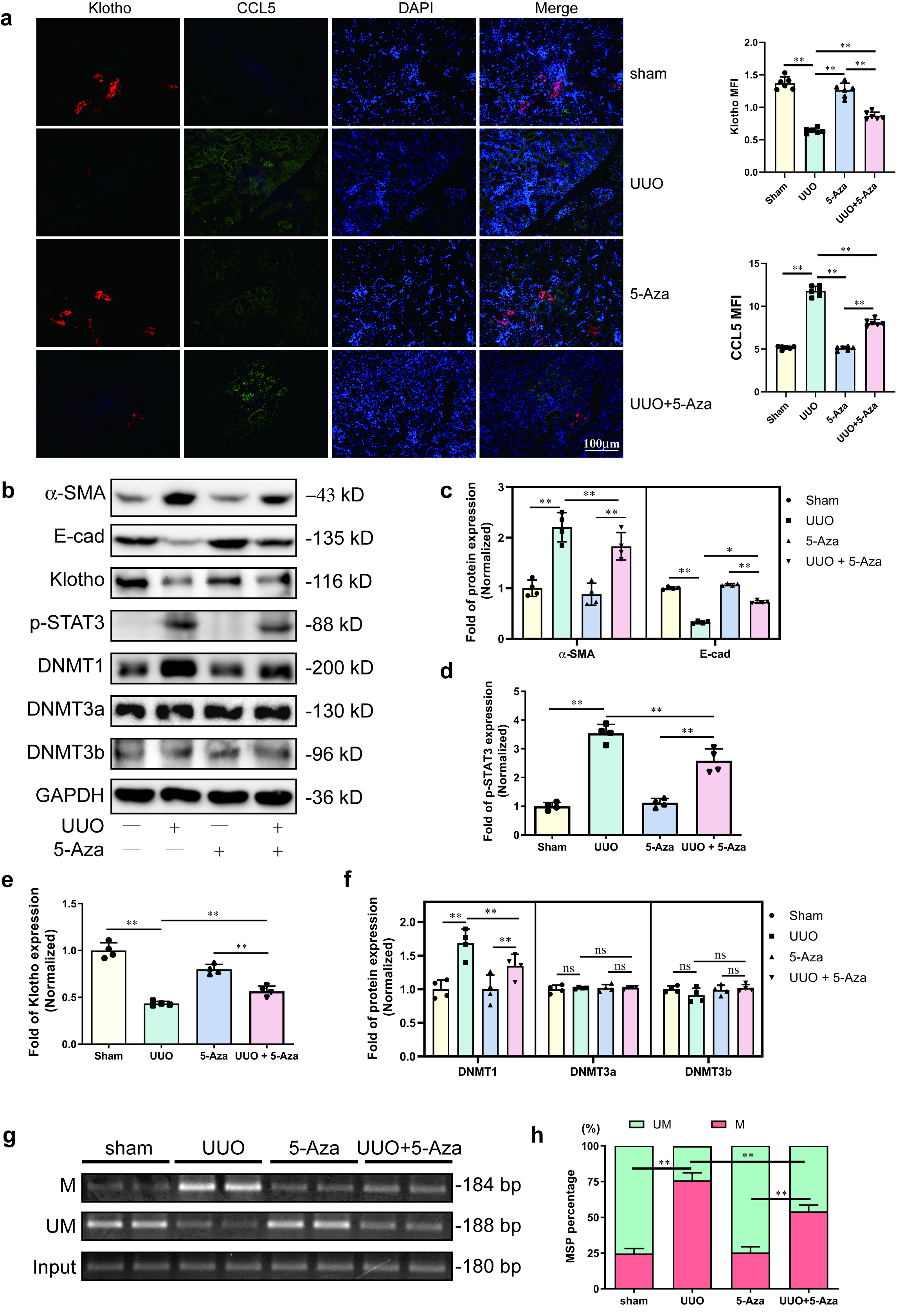

Supplement: Supplementary file 5 [file Image_3.tif]

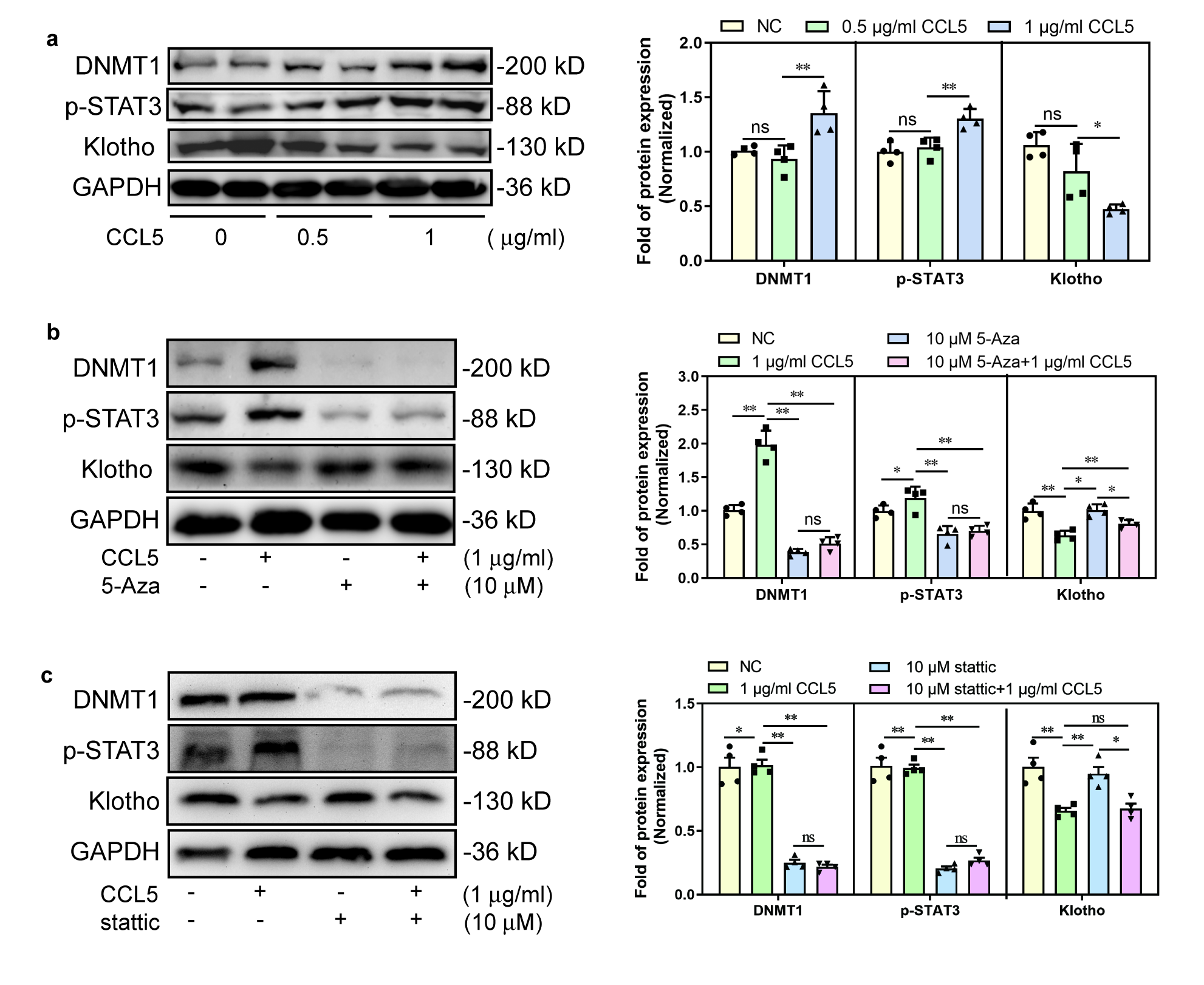

Supplement: Supplementary file 6 [file Image_4.tif]
